# Microlithic variation and the Mesolithic occupations of western India

**DOI:** 10.1371/journal.pone.0267654

**Published:** 2022-06-22

**Authors:** Charusmita Gadekar, Juan José García-Granero, Marco Madella, P. Ajithprasad

**Affiliations:** 1 HUMANE–Human Ecology and Archaeology, Department of Archaeology and Anthropology, Milà i Fontanals Institution for Research in Humanities (IMF), Spanish National Research Council (CSIC), Barcelona, Spain; 2 School of Archaeology, University of Oxford, Oxford, United Kingdom; 3 CaSEs, Department of Humanities, Universitat Pompeu Fabra, Barcelona, Spain; 4 Institució Catalana de Recerca i Estudis Avançats (ICREA), Barcelona, Spain; 5 School of Geography, Archaeology and Environmental Studies, The University of the Witwatersrand, Johannesburg, South Africa; 6 Department of Archaeology and Ancient History, The M.S. University of Baroda, Vadodara, India; University of Edinburgh, UNITED KINGDOM

## Abstract

Considerable confusion and uncertainty persist on the cultural and chronological contexts of Holocene microlithic assemblages reported from South Asia. The paucity of securely dated sites with microlithic remains has compounded the confusion. Evidence from sites securely attributed to the Mesolithic based on a holistic approach (including direct evidence of plant and animal exploitation strategies) is needed to provide a better understanding of Mesolithic lithic tool-kits. This study uses morphometric and statistical methods to assess the nature of the Holocene hunter-gatherer microlithic tools-kit from a radiometrically secured chronological context at Vaharvo Timbo, a recently excavated Mesolithic site in North Gujarat (India). The assemblage is further compared with the nearby contemporary site of Loteshwar to highlight similarities and differences within hunter-gatherer lithic assemblages, understanding which can provide detailed information about subsistence strategies as well as patterns of settlement and mobility. The results show general standardisation between these two sites regarding raw materials and manufacturing technique but variation in the relative abundance of tool types between these two sites, despite their close proximity, indicating diverse strategies of resource exploitation by the Holocene hunter-gatherer groups in western India.

## Introduction

Microliths are small retouched stone tools, usually considered to be hafted as part of composite tools [1]. Despite attempts spanning several decades, an accepted standard definition of microliths has failed to materialise [2–7]. Existing definitions often use size limits and require the use of microblade blanks, geometric shapes and/or backing retouch [8]. From their first discovery by A. C. Carlleyle in the Vidhya Hills of Central India in 1867 (Carlleyle´s notes quoted in [9]), microlithic assemblages have been found across much of the world. Microlithic industries are particularly common in the Holocene, but a trend towards frequent microlith production has been noted in the Late Pleistocene all over the world [1]. The technological details of microlithic artefacts vary widely; nevertheless, the fact remains that by 10 kya (thousand years ago) foraging groups on every inhabited continent were regularly producing and utilising large number of very small tools [10]. In South Asia, Late Palaeolithic microlithic technologies begin to appear in the archaeological record from ca. 45 kya [11, 12] and remain in use by hunter-gatherer populations for much of the Holocene, when they are referred to as Mesolithic industries [13].

In India, the beginning of the Holocene, which witnessed the commencement of a climatic amelioration, is related with sophisticated techniques of stone tools manufacturing that are associated with the Mesolithic period [14]. This post-glacial period has often been presented simply as a chronological disjuncture from what happened before (the Palaeolithic) and what would follow (the Neolithic, a period which saw the first sustained use of domesticated plants and animals) [15].

The Mesolithic period in India is characterised by semi-nomadic human populations who carried out hunting, gathering, foraging and fishing. There was a marked growth in human population, as attested by the significantly increased number of sites [16]. The first human colonisation of the Ganga plains, deltaic region of West Bengal and West coast and Kerala took place during this period [17–21]. All daily household activities were being carried out with the help of microlithic toolkits [22], which have been reported from all over the subcontinent [11, 23–32]. Microliths thus historically have been considered diagnostic tools to chronologically attribute a site to Holocene hunter-gatherer groups [33]. However, it has become apparent that there was no single industry or technology exclusively for the Holocene hunter-gatherers and even though microlithic technology might have identified as the diagnostic character of the Mesolithic period, it is not synonymous to Mesolithic [34]. Regional variability can be seen commonly [11, 25–31, 35, 36]. Moreover, microliths have been documented from a wide range of chronological and archaeological contexts [35, 36], and thus defining a site based solely on the presence of these tools is flawed, especially when it comes to the Holocene period. Regardless, archaeologists working in the subcontinent still associate microliths with the Mesolithic period alone and tend to label all microlithic scatters encountered through surveys as ‘Mesolithic’ irrespective of their uncertain chronology and their possible contemporaneity with Neolithic, Chalcolithic or Iron Age deposits. It is time that a clearer criterion for the systematization of South Asian microliths of different ages distinguished on the basis of shape, dimension, type of retouch and technology is established and followed.

Microlithic scatters are present virtually on every one of the thousands of relic dunes in the arid and semi-arid regions surrounding the Thar Desert, including present-day Sindh in Pakistan [37] and western Rajasthan and North Gujarat in India [16]. These deposits are normally sitting at the top of sand dunes in front of saltwater and freshwater basins of seasonal rivers [38–45]. Sedimentological andisotopic analyses from the lacustrine sediments of salt lakes in Rajasthan showed that these basins began to form just before the beginning of the Holocene, when the dunes were still active [46–49]. Independently of their origin, the level of these lakes increased at first in the Early Holocene, as shown by radiocarbon dates obtained from the lower sediment deposits of Lake Sambhar (TF-887: 9250±50 BP) and Lukaransar (Sio 405: 9260±115 BP). This moment marks an increase in rainfall and a wetter climate [49, 50], which led to an increase in vegetation and the stabilisation of the dunes. Preliminary data from interdunal depressions from North Gujarat suggest that perennial water bodies existed until ca.7 kya [51]. Such interdunal depressions retained water throughout most of the year and acted as *foci* of attraction for the hunter-gatherer groups, most probably due to the presence of ungulates approaching the water bodies for drinking [52]. The discovery of many hunter-gatherer deposits at the top of these dunes throughout the Thar Desert and surrounding regions indicates that many areas of this desert, from its periphery to the central part, began to stabilise at the beginning of the Holocene. At the same time, the stratigraphy of the occupations in northern Gujarat, with deposits between 40 and 150 cm in depth, indicates that some areas/dunes were active beyond the beginning of the Holocene. However, the absolute chronology of most of the reported sites is still unknown [36], partly due to the absence of a developed stratigraphy, which might easily result in contamination and the impossibility to carry out absolute chronology determinations on the deposits. Even though the sites from Upper and Lower Sindh show that the hunter-gatherers of the Early Holocene temporarily settled environmentally different landscapes, absence of datable organic materials have forced their chronology to be established only through the typological analysis of lithic assemblages and their comparison with those of radiocarbon-dated sites in India [37]. For this reason, settlements in North Gujarat, which have deeper stratigraphy, are important to understand the temporality and nature of such hunter-gatherer occupations in this arid area.

Patel [53] noted that dune-top settlements in North Gujarat and southern Rajasthan have complicated depositional histories that can be disentangled only through the use of a combination of techniques that include well-designed dating protocols together with micro-depositional studies with a focus on individual activity areas and episodes of occupation. Moreover, defining an archaeological deposit as the remains of activities conducted by hunter-gatherer groups should not rely exclusively on the kind of artefacts present at the site but should also consider the direct evidence of the food-related activities being conducted by these groups: plant and animal remains. Unfortunately, archaeobotanical and zooarchaeological analyses have been seldom conducted in hunter-gatherer occupations in North Gujarat and southern Rajasthan [for exceptions see e.g. 54, 55], thus limiting our capability of studying lithic assemblages from unequivocally hunter-gatherer deposits.

The aim of this paper is to analyse the lithic assemblage from Vaharvo Timbo, a recently excavated and thoroughly studied hunter-gatherer occupation in North Gujarat [51, 56, 57], to assess the nature of Holocene hunter-gatherer lithic tool-kits in western India. The site has been securely radiocarbon dated and does not show the existence of either domesticated animals or plants. The lithic assemblage from Vaharvo Timbo is further compared with the hunter-gatherer deposits of the nearby contemporary site of Loteshwar [51, 53–61]. Loteshwar is one of the most systematically investigated sites of North Gujarat, incorporating both Mesolithic (hunter-gatherer) and Chalcolithic (Anarta Chalcolithic, food producers) cultural layers. This comparison shows what hunter-gatherer lithic assemblages comprise of in this region, can be used as a reference for future research in western India and Sindh and allows us to understand variability within microlithic toolkits of hunter-gatherer occupations within the same ecological settings.

## Case study

**Fig 1:**
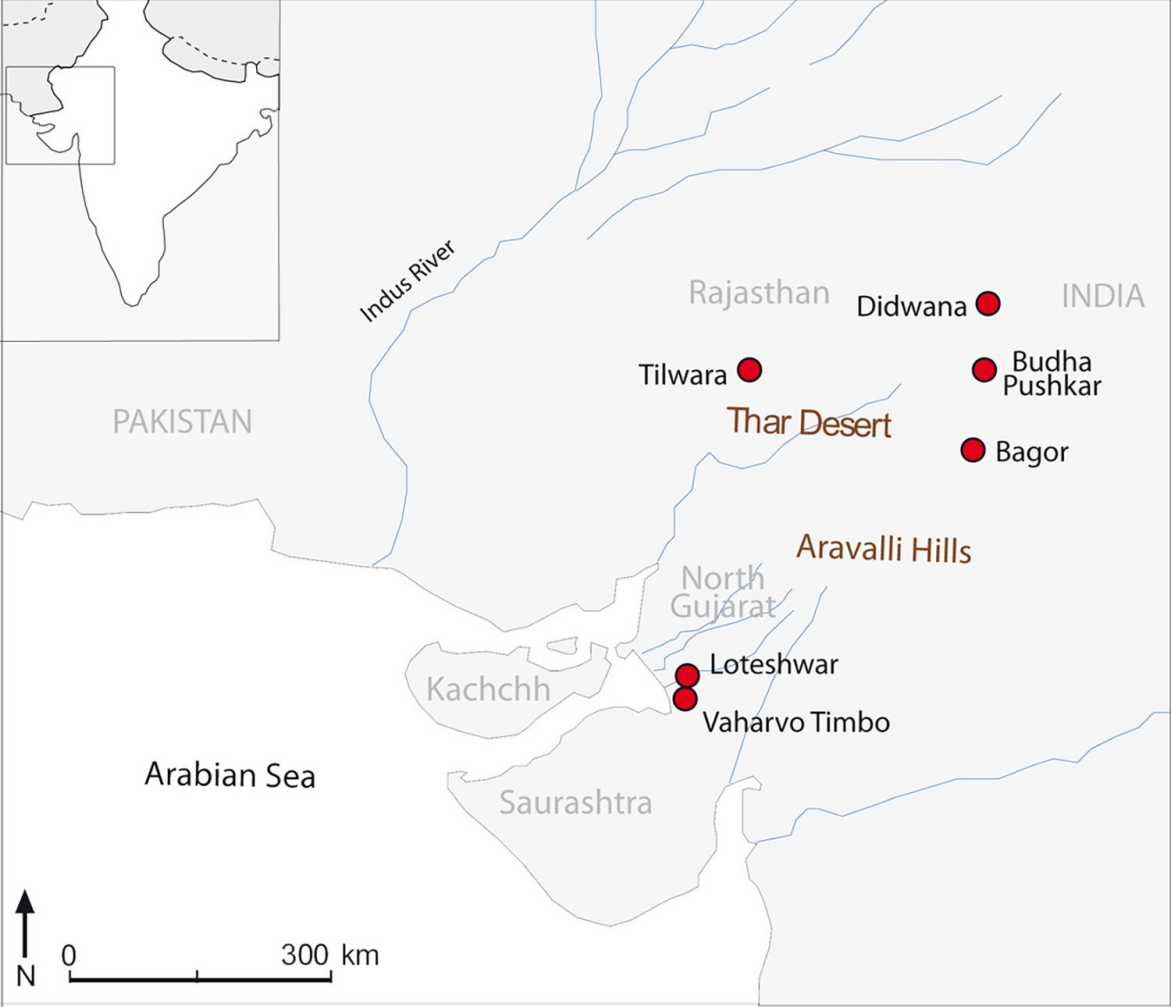
Map of western India showing the location of the archaeological sites mentioned in the text. Background figure courtesy of Conesa FC (North Gujarat Archaeological Project).

Vaharvo Timbo (23^0^33’17.5”N, 71^0^48’12.01”E) is situated in the Runi village, Sami Taluka of Patan district of Gujarat, India (Fig 1). It was reported in the 1980s as Wasar no timbo [62]. The site measures 5.2 ha and is located at the summit of a set of stabilised sand dunes in the estuary of the Rupen river, which drains into the Little Rann of Kachchh. The site was excavated by the North Gujarat Archaeological Project (NoGAP), a collaboration between The Maharaja Sayajirao University of Baroda, Vadodara, Gujarat, India and the Institució Milà i Fontanals, Spanish National Research Council, Barcelona, Spain [61]. Two 4x4m trenches were excavated in 2011, one near the top of the dune and the second further down the slope. The purpose was to understand the expansion and depth of the microlithic deposit of this dune and the relationship between artefacts and bioarchaeological remains. Trench 1 showed a uniformly aceramic hunter-gatherer occupation down to 135 cm, with three pits of different size and shape. The excavated artefacts include lithic tools and debitage, pieces of ochre, iron or manganese oxide nodules, *Dentalium* sp. shell beads, bone points and grinding stones [57], and it was AMS-dated to c. 5600–5000 cal. BC [51]. Trench 2 was also an aceramic deposit cut by a human burial. The inhumation burial contained the skeleton of a young individual in association with two pottery vessels, both showing Early Harappan (c. 3300–2600 BC) characteristics [63].

Loteshwar (23^0^36’1.8”N, 71^0^50’11.8”E) is situated about 6 km from Vaharvo Timbo (Fig 1) and was re-excavated under the NoGAP team in 2009 [64]. The Mesolithic deposits have been AMS dated to 7168–4703 cal. BC and it is one of the earliest Holocene hunter-gatherer occupations in north-western India [53, 65–67]. The Mesolithic lithic assemblage from the 2009 excavation at Loteshwar, which has already been published in detail [59], comprises of 402 blades (simple blades n = 164, backed blades n = 28, blade flakes n = 184, retouched blades n = 23 and obliquely blunted blades n = 3), geometric and non-geometric/ tools such as isosceles triangles and scalene triangles (n = 8), lunates (n = 45), trapezes (n = 8) and points (n = 11). The lithic debitage includes flakes (primary flakes, secondary flakes and core rejuvenation flakes n = 1202), nodules (n = 16), waste/shatter (n = 289) and cores (blade cores n = 46 and flake cores n = 9).

Vaharvo Timbo and Loteshwar are among the few recently excavated hunter-gatherer sites in western India where controlled excavation methods were applied together with a comprehensive sampling methodology for geo- and bio-archaeological techniques so that a broad dataset was collected from the anthropic deposits [51, 57, 59, 64, 68, 69], also making the archaeological assemblages directly comparable. These included charred plant macroremains, wood charcoal, animal bones, phytoliths and starch as well as sediments analyses and micromorphology. Moreover, extensive geoarchaeological and vegetation surveys were conducted to reconstruct regional landscape dynamics [70, 71], while remote sensing and satellite surveys were conducted to identify physiographic features in a multi-scale perspective [52, 71–73].

Published zooarchaeological data from Loteshwar and the on-going faunal analysis from Vaharvo Timbo suggest that the inhabitants of these sites hunted small wild bovids, especially antilopines (mostly *Antilope cervicapra*) [50; O. Parque personal communication]. Moreover, the archaeobotanical record from Vaharvo Timbo suggests gathering of wild grasses and the possible exploitation of wild palms as well as other plants from the more humid interdunal areas. The plant remains recovered from Loteshwar show that the hunter-gatherer exploited woody plants (high presence of phytoliths from dycotiledoneous plants as well as wood charcoal) and also collected panicoid grasses and wild pulses for carbohydrates [51]. Overall, the archaeobotanical data shows that a wide range of plants originating from semi-permanent water bodies were being exploited.

## Materials and methods

The aim of this study is to assess the nature of the lithic assemblage from a purely hunter-gatherer occupation, thus only the artefacts from Trench 1 from Vaharvo Timbo have been considered in this study. The lithic assemblage was first classified by raw materials. Further classification of the lithic assemblage into finished tools (retouched into preconceived shapes) and lithic debitage (the manufacturing waste) was done according to their morphological features. All the tools were classified into 1) various types of blades which includes simple blades (parallel sided blades without any retouches but with edge damage), backed blades (blades with retouches along one longitudinal side), obliquely blunted blades (with oblique retouches on one of the traverse side) and retouched blades (with random retouches); 2) geometric tools such as isosceles tringles (two equal sides), scalene triangles (without equal sides), lunates (shaped like a crescent) and trapezes (two parallel horizontal sides and two non-parallel shorter sides, the non-parallel or the transverse sides are retouched); and 3) non-geometric tools such as points (with a pointed end which is achieved by retouching the surface all around) and double sided scrapers (flake with retouches on two longitudinal sides)—please refer to [59] for detailed information about tool classification methodology. Length, breadth and thickness of all the tools were measured with a digital calliper to explore potential standardisation in tool manufacturing. Tool edges were analysed macroscopically and unintentional edge damages were taken as evidence of utilisation. The rationale is that tools will show edge damage (not intentional retouches) only if they have been utilised. Metric data from Vaharvo Timbo were summarised in tables and expressed as boxplots for visualisation. The statistical significance of the differences of the size of the tools from Vaharvo Timbo and Loteshwar was tested using a Mann-Whitney-Wilcoxon test—a non-parametric test was chosen because most data are non-normally distributed. All the statistical analyses were computed using the software R 3.4.2 on RStudio 1.1.383 using the packages ‘ggplot2’ and ‘reshape2’ for plotting the boxplots. The raw metric data and the outcome data of the statistical analyses can be found in S1 Table and S1 Appendix, respectively.

## Results

**Fig 2:**
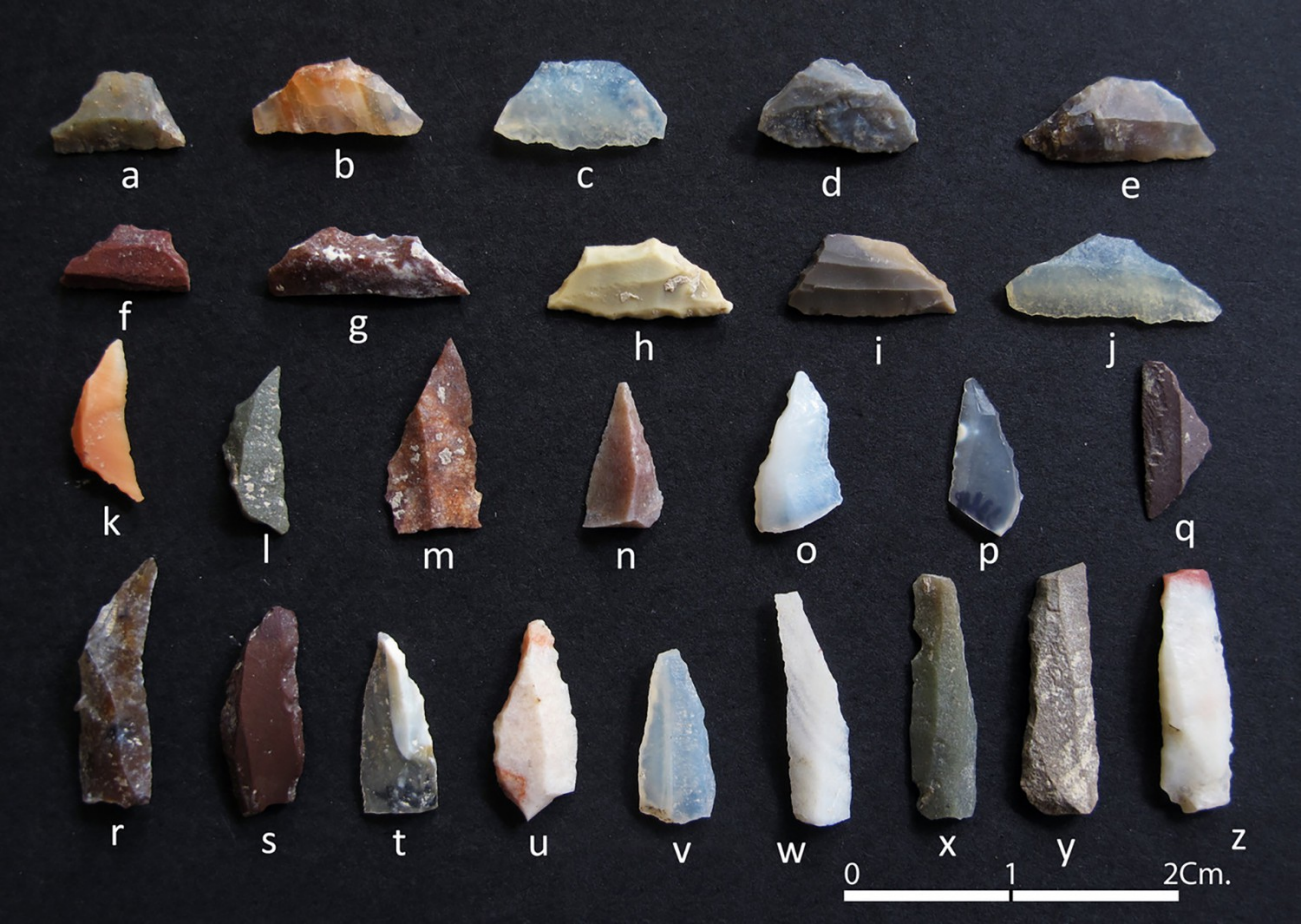
Examples of the lithic assemblage recovered from Vaharvo Timbo: a-j) trapezes, k-l) lunates, m-p) points, q) triangle isosceles and r-z) various types of blades. Courtesy of the Department of Archaeology and Ancient History, The M.S. University of Baroda, Vadodara. Photo credits: Ajithprasad P (North Gujarat Archaeological Project).

The lithic assemblage from Vaharvo Timbo Trench 1 comprises 192 blades, 90 geometric tools, 20 non-geometric tools and 5034 fragments of lithic debitage, including exhausted cores (Fig 2). Raw materials belong to different types of crypto-crystalline siliceous materials including chert, chalcedony, banded agate, moss agate, carnelian, bloodstone and quartz. A few fragments of mica and quartzite (not involved in tool production) were also identified from the assemblage.

### The blade assemblage

The total of 192 blades identified were further classified according to their attributes into simple blades (61.14%), backed blades (16.06%), obliquely blunted blades (11.91%) and retouched blades (10.88%). The majority of the blades were made out of chert (47.52%), followed by chalcedony (26.40%), banded agate (11.22%) moss agate (9.24%), quartz (4.62%) and carnelian (0.99%). Most of the blades were found in broken condition (86.53%) and have been further classified according to their fracture into proximal (34.72%), mesial (35.75%) and distal fragments (8.81%). Most of the blades (90.10%) did not show presence of cortex. Since secondary blades are intentionally selected to manufacture specialised tools, it was observed that all the retouched blades were devoid of cortex.

**Fig 3:**
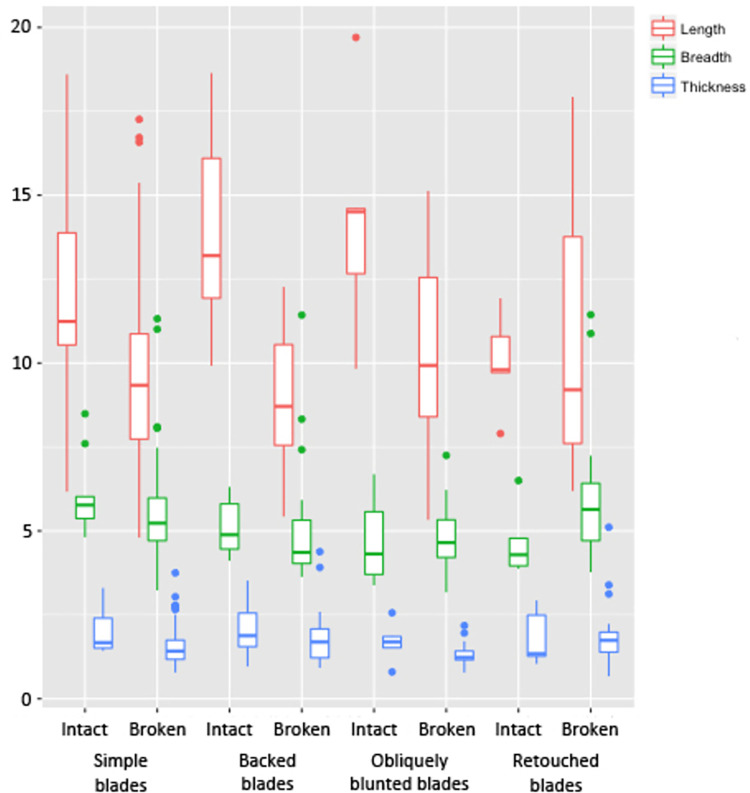
Boxplot showing the length, breadth and thickness (in mm) of the blades from Vaharvo Timbo.

Summarised metric data from blades is shown in Table 1 and Fig 3. Blade flakes were not included in the analysis due to the low number of artefacts in this category (n = 3). Complete blades are low in numbers (n = 5–9 in all categories) but they have been included in the statistics to be compared with broken blades of similar categories. Simple blades, which do not display any form of retouches but show utilisation in the form of edge damage (macroscopic examination), do not show much variation in their sizes. Backed blades, which were intentionally chipped to facilitate hafting as well as to increase the amount of force that could be applied to the worked material [74], show variation only in their length values; hardly any variation is seen in their breadth and thickness values.

**Table 1:** Summarised metric data (in mm) of the blades from Vaharvo Timbo.

			Mean	Median	Mode	Std Dev	Min.	Max.
Simple blades	Complete blades (9)	*Length*	12.44	11.24	6.17	3.9	6.17	18.6
		*Breadth*	6.05	5.77	4.81	1.22	4.81	8.49
		*Thickness*	2.06	1.67	1.43	0.68	1.43	3.3
	Broken blades (108)	*Length*	9.58	9.37	10.73	2.68	4.8	17.26
		*Breadth*	5.44	5.24	5.26	1.29	3.23	11.32
		*Thickness*	1.5	1.41	1.01	0.54	1.1	3.75
Backed blades	Complete blades (6)	*Length*	13.92	13.2	9.92	3.29	9.92	18.64
		*Breadth*	5.1	4.88	4.11	0.89	4.11	6.31
		*Thickness*	2.07	1.88	0.96	0.92	0.96	3.52
	Broken blades (25)	*Length*	8.95	8.71	5.43	1.85	5.43	12.27
		*Breadth*	5.06	4.36	3.63	1.74	3.63	11.43
		*Thickness*	1.82	1.69	0.92	0.85	0.92	4.38
Obliquely bunted blades	Complete blades (5)	*Length*	10.03	9.8	7.9	1.49	7.9	11.93
		*Breadth*	4.68	4.29	3.87	1.08	3.87	6.5
		*Thickness*	1.81	1.34	1.04	0.84	1.04	2.93
	Broken blades (16)	*Length*	10.48	9.21	6.19	3.74	6.19	17.93
		*Breadth*	6.09	5.64	3.77	2.21	3.7	11.44
		*Thickness*	1.98	1.74	0.67	1.07	0.67	5.11
Retouched blades	Complete blades (5)	*Length*	14.21	14.51	14.51	3.29	9.83	19.7
		*Breadth*	4.69	4.32	3.38	1.32	3.38	6.69
		*Thickness*	1.68	1.69	0.8	0.57	0.8	2.56
	Broken blades (17)	*Length*	10.52	9.93	5.33	2.87	5.33	15.13
		*Breadth*	4.77	4.65	4.21	0.97	3.17	7.25
		*Thickness*	1.35	1.23	1.14	0.35	0.78	2.18

Obliquely blunted blades, also known as penknife blades [75] due to their shape, appear to have been hafted and might have been used as a point for arrow/lance or barbs for harpoons. There is a difference in the length of these blades between complete blades and broken blades, but not in their breadth and thickness. Finally, the length of retouched blades, which have random retouches on their lateral edges most probably produced for hafting purposes, shows little variation despite the fact that most of the blades were found in broken condition.

### Geometric and non-geometric tools

Isosceles (18.88%) and scalene triangles (34.44%), lunates (34.44%) and trapezes (12.22%) form the categories of geometric tools recovered at the site. The majority of the tools were found in intact condition. The tools which were not complete showed minor breakages (e.g., a tiny portion of the tip was broken), thus all the tools have been taken together for their metric analysis (Table 2). 97.27% of the geometric tools were made from secondary blades. Interestingly, almost half of the tools seem to have not been utilised (macroscopic examination). Metric details suggest that the geometric tools were made from similar types of blades, as they appear to have had similarity in their length, breadth and thicknesses (Table 2).

**Table 2:** Summarised metric data (in mm) of the geometric and non-geometric tools from Vaharvo Timbo.

	Attributes	Mean	Median	Mode	Std Dev	Min.	Max.
Triangles Isosceles (17)	*Length*	10.40	10.34	6.84	2.16	6.84	16.85
	*Breadth*	4.86	4.93	4.93	0.63	3.65	6.2
	*Thickness*	1.78	1.78	1.00	0.62	1.00	2.98
Triangles Scalene (31)	*Length*	10.06	9.82	7.53	2.22	6.78	16.96
	*Breadth*	5.13	5.08	5.08	0.83	3.38	6.76
	*Thickness*	1.82	1.61	1.54	0.53	1.02	2.95
Lunates (31)	*Length*	11.89	11.47	5.52	3.24	5.52	19.00
	*Breadth*	5.31	5.09	4.58	1.04	3.7	8.03
	*Thickness*	2.30	2.12	1.3	0.72	1.3	3.98
Trapezes (11)	*Length*	10.89	10.67	7.84	2.00	7.84	14.82
	*Breadth*	4.91	4.85	3.91	0.70	3.91	6.3
	*Thickness*	1.82	1.70	1.06	0.69	1.06	3.74
Points (16)	*Length*	10.66	10.45	7.2	2.27	7.2	16.44
	*Breadth*	4.81	4.90	3.43	0.755	3.43	6.05
	*Thickness*	2.08	1.98	1.15	0.85	1.15	4.81

**Fig 4:**
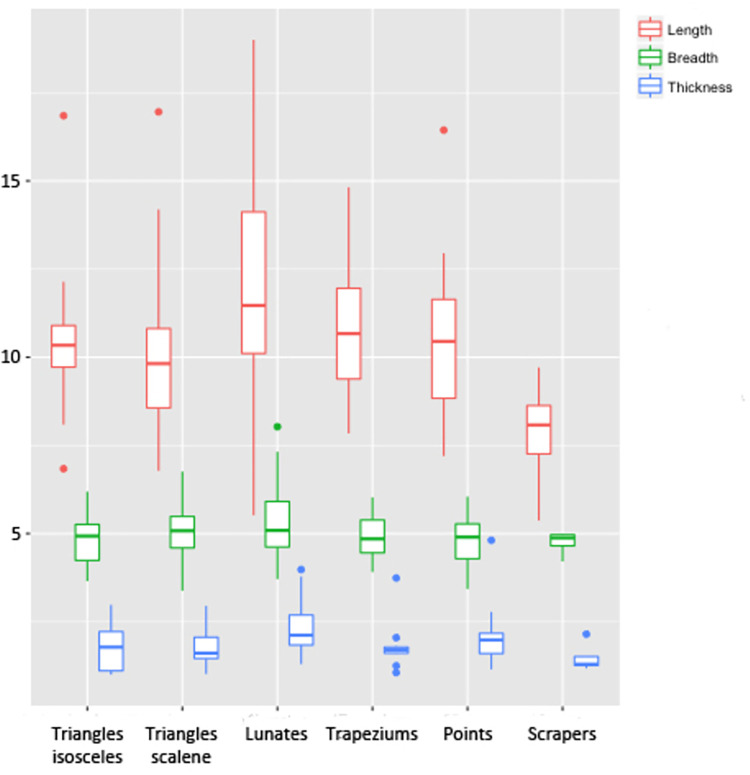
Boxplot showing the length, breadth and thickness (in mm) of the geometric and non-geometric tools from Vaharvo Timbo.

Non-geometric tools include points (n = 16) and double-sided scrapers (n = 4). Around 70% of the points were found broken. However, a separate metric analysis of complete and broken points did not show significant change in their values, and thus a combined analysis is presented here (Table 2 and Fig 4). Points appear to follow the same pattern in their metric measurements as the geometric tools. The low number of scrapers did not allow for a separate metric analysis. Chert was the favoured material to manufacture both geometric as well as non-geometric tools, followed by chalcedony, banded agate and moss agate. A few artefacts were also made from quartz, carnelian and bloodstone.

### Lithic debitage

Lithic debitage represents the discarded and unused detached pieces of lithic material produced from the reduction of an objective piece [74]. These by-products of stone tool productions have been classified into primary flakes (n = 1302 or 25.86% of total debitage), secondary flakes (n = 2119 or 42.09%), core rejuvenation flakes (n = 5 or 0.09%), nodules (n = 70 or 1.39%), cores (n = 152 or 3.02%) and waste/shatter (n = 1386 or 27.53%). Amongst the raw materials, chert predominates (38.43%), followed by chalcedony (23.78%), banded agate (19.52%), quartz (10.30%), moss agate (3.42%), carnelian (3.50%) and quartzite (0.72%).

**Fig 5:**
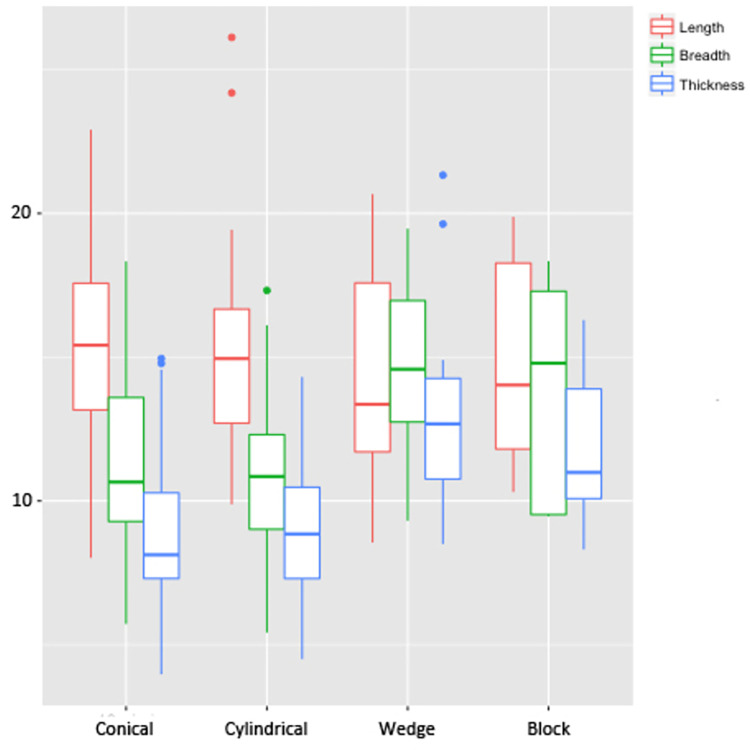
Boxplot showing the length, breadth and thickness (in mm) of the blade cores from Vaharvo Timbo.

The presence of exhausted blade cores, core rejuvenation flakes and lithic debitage clearly shows that the tools were manufactured at the site. The majority of cores belong to chert (54.3%), followed by banded agate (21.19%), chalcedony (13.25%), quartz (5.96%), moss agate (3.31%), blood stone (0.66%) and carnelian (1.32%). Only one flake core has been identified from the assemblage. The blade cores have been classified into conical (56.95%), cylindrical (28.48%), wedge (9.93%), block (3.31%) and amorphous (1.32%) shapes according to their morphological features. Conical and cylindrical shapes of cores are formed due to unidirectional removal of blades and are associated with end result of blade technology, whereas wedge-shaped cores are formed when a nodule is quartered into pieces and then flaked from one surface [36] and block cores have a cubical look since blades are removed from all sides. In spite of the differences in their shapes, the blade cores show similarity in their sizes (Table 3 and Fig 5). Low numbers of amorphous cores (n = 2) did not allow for statistical analysis.

**Table 3:** Summarised metric data (in mm) of the blade cores from Vaharvo Timbo.

		Mean	Median	Mode	Std Dev	Min.	Max.
Conical (86)	*Length*	15.32	15.25	10.27	3.16	8.02	22.91
	*Breadth*	11.72	11.17	9.62	3.02	5.72	18.33
	*Thickness*	9.22	8.77	7.35	2.64	3.97	14.95
Cylindrical (43)	*Length*	15.39	15.04	10.59	3.55	9.88	26.12
	*Breadth*	11.35	11.13	8.69	2.80	5.42	17.32
	*Thickness*	9.52	9.11	8.69	2.85	4.5	14.31
Wedge (15)	*Length*	13.95	13.36	8.55	3.31	8.55	20.67
	*Breadth*	13.84	13.89	6.85	3.41	9.31	19.47
	*Thickness*	12.77	12.53	8.50	3.58	8.5	21.33
Block (7)	*Length*	15.69	16.17	10.31	3.29	9.7	19.88
	*Breadth*	14.2	15.99	7.59	4.62	9.46	18.34
	*Thickness*	12.38	12.17	8.31	3.26	8.31	16.29

Blade removal pattern of cores shows that 82.78% cores were rotated to remove blades from them. More than 90% cores showed unidirectional blade removal. It was observed that 47.02% cores showed removal of a single flake for platform preparation while 43.71% core platforms were prepared by removing multiple tiny flakes from the surface. Only 36.42% cores showed complete absence of cortex.

### Comparison with Loteshwar

**Fig 6:**
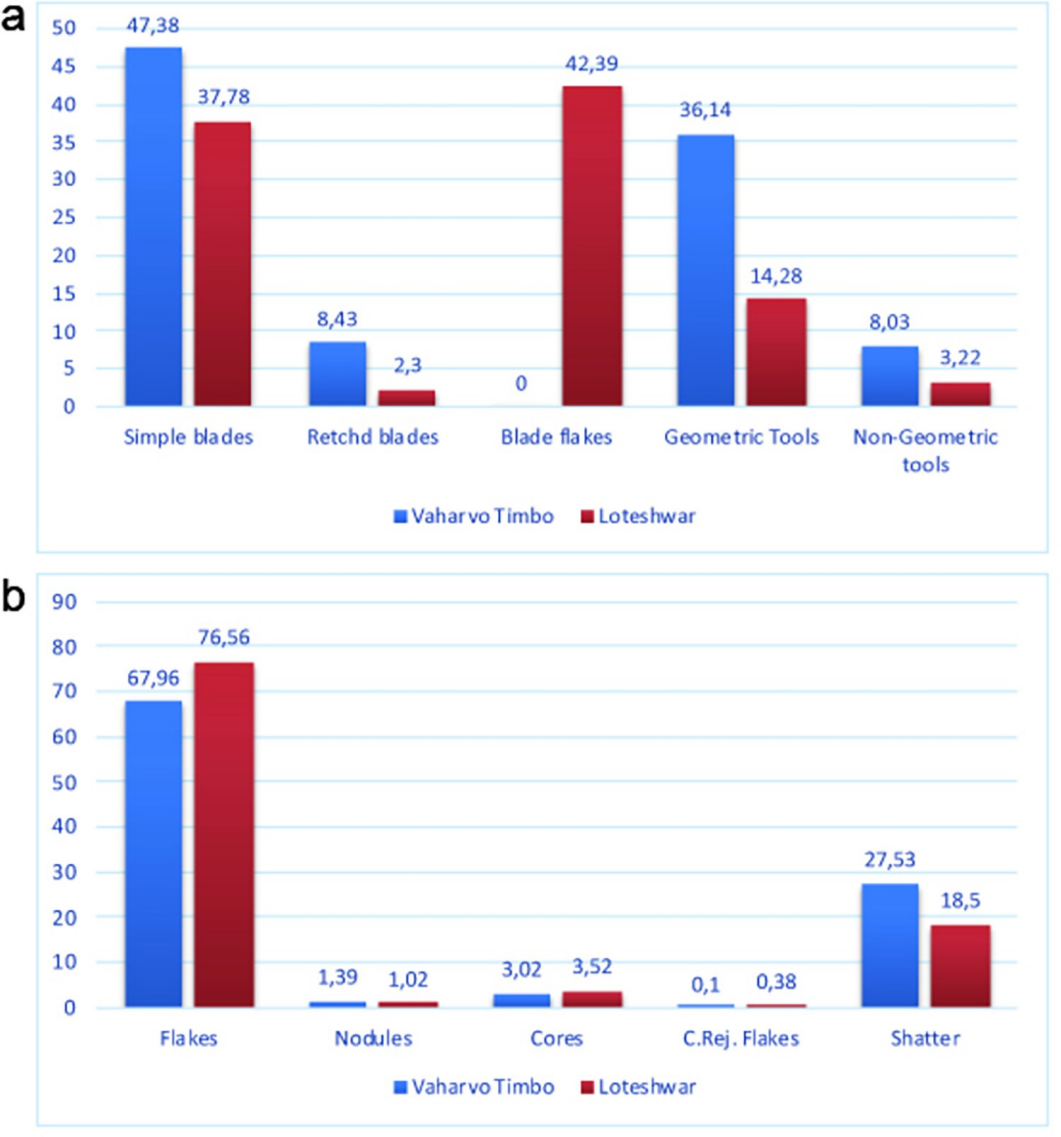
Comparison (in percentages) of the lithic assemblages from Loteshwar and Vaharvo Timbo: a) blades and tools, b) lithic debitage.

The lithic assemblages from Vaharvo Timbo and Loteshwar present comparable tool kits, albeit with some striking differences (Fig 6). The raw materials exploited and the techniques used to manufacture the tools at both sites have been found to be the same. The preponderance of blade cores at both sites (n = 151 from Vaharvo Timbo and n = 46 from Loteshwar) affirms the presence of formal tools such as blades and geometric tools made out of blades. However, the comparison of the lithic assemblages also shows important variability. Retouched blades (backed blades, n = 31, and obliquely blunted blades, n = 21) and geometric tools (triangles, n = 48, trapezes, n = 11, and lunates, n = 31) are present in higher numbers at Vaharvo Timbo, whereas blade flakes (flakes without uniform parallel sides like a blade), virtually absent from Vahavo Timbo, are abundant (n = 112) at Loteshwar.

The statistical comparison of the metric data from the blades, geometric tools and cores from Vaharvo Timbo and Loteshwar shows that there are no statistically significant differences between the assemblages from both sites for most measured variables (Table 4). Regarding blades, statistically significant differences were found in the breadth of simple blades, backed blades and obliquely blunted blades—note that the analysis excluded blade length because most blades were found broken at both sites. Regarding geometric and non-geometric tools, statistically significant differences were found in all three measured variables from lunates and in the length of scalene triangles, although the latter might be a result of the small number of individuals found at Loteshwar (n = 3). Finally, statistically significant differences were also found in the breadth and thickness of wedge cores from Vaharvo Timbo and Loteshwar.

**Table 4:** Results of the Mann-Whitney-Wilcoxon tests (p-values) comparing the median and standard deviation of metric data from blades, tools and cores from Vaharvo Timbo and Loteshwar.

	Length	Breadth	Thickness
*Blades*			
Simple blades	–	**0.01**	0.17
Backed blades	–	**0.001**	0.29
Obliquely blunted blades*	–	**0.04**	0.37
Retouched blades	–	0.23	0.37
*Tools*			
Triangles isosceles	0.25	0.55	0.83
Triangles scalene*	**0.03**	0.67	0.54
Lunates	**0.03**	**<0.001**	**<0.05**
Trapezes	0.28	0.78	0.13
Points	0.16	0.49	0.68
*Cores*			
Conical	0.34	0.73	0.60
Cylindrical	0.88	0.10	0.85
Wedges	1	**0.03**	**<0.001**
Blocks*	0.93	0.65	0.31

Significant differences indicating that the assemblages from the two sites are statistically different are highlighted in bold. Asterisks (*) indicate that the number of individuals in one of the groups is below five and thus the results are not statistically reliable.

## Discussion and conclusion

Microliths have been conventionally characterised in terms of size and shape. It has been observed that most of the specimens are small, sometimes very small (average lengths ranging from one to five cms [16]), and yet often standardised and precisely made relative to other classes of stone tools [7]. The retouching of geometric tools is aimed at transforming a blade into an efficient tool by blunting a natural sharp border of the blade. Commonly thought to have been used as projectile tips, arrow points, barbs and side inserts [76–85], these tools can also be used for other functions, especially to make composite tools such as sickles or scythe [86–88]. Although Mesolithic and Chalcolithic assemblages in India incorporate varieties of geometric and non-geometric tools, starting from the middle of the 8^th^ until the end of the 2^nd^ millennium BC, a peruse of the lithic assemblages clearly shows that sites do display a preference for one or the other type of tool [1, 35]. Why certain tools were preferred at certain sites is a question that needs further exploration.

The inhabitants at Vaharvo Timbo were involved in hunting, gathering, foraging and fishing activities using microliths. The lithic assemblage is composed of various types of blades, geometric and non-geometric tools and lithic debitage. The large amount of lithic debitage as well as the presence of exhausted cores suggest on-site manufacturing of these tools. The site was devoid of ceramics but archaeobotanical remains suggest a diverse exploitation of plants, including four different species of grasses, palms and sedges and wild sesame seeds [51]. Evidence from on-going zooarchaeological analyses shows that people were hunting small wild bovids, especially antilopes (O. Parque, personal communication). Most of the tools at Vaharvo Timbo did not show macroscopic evidence of edge damage leading to the question of why people would invest time and energy to manufacture such tools but then did not actually use them. Can this behaviour be taken as evidence of future planning? The inhabitants at Vaharvo Timbo were clearly involved in hunting activities as shown by the abundant faunal remains. It has been suggested that it is easy to lose tools/parts of a tool while performing various activities. Geometric tools, mostly thought to be used for hunting activities, might have been produced, some of them used and some kept by the inhabitants for whenever necessary.

The statistical analysis revealed that the size of microliths from Vaharvo Timbo and Loteshwar show a high degree of standardisation, suggesting that similar techniques werer utilised to manufacture the tools at both the sites. In spite of this, when the lithic data from Vaharvo Timbo and Loteshwar is integrated with zooarchaeological and archaeobotanical data, it shows that activities conducted at each site varied. Loteshwar has given evidence of exploitation of wild animals in terms of blackbuck, gazelle, boar, wild cattle, wild water buffalo, nilgai, hemione and two forms of deer [53]. While the archaeobotanical evidence shows high presence of dicot phytoliths, suggesting hunter-gatherers exploited woody plants more along with exploitation of several species of panicoid grasses and wild pulses [51].

Differences in tool-kits between sites can result from a variety of reasons, including the technique used to produce them, specific chrono-cultural settings or the availability of raw materials. In this case, however, Loteshwar and Vaharvo Timbo’s tool assemblages are comparable in all these aspects. The higher presence of geometric tools at Vaharvo Timbo might be due to the fact that the main activity of the people camping here was hunting, which most probably required the use of more geometric tools, whereas the more varied lithic tool-kit recovered at Loteshwar might reflect a more diverse set of activities being carried on at the site. Loteshwar is located about 500 m east of the Khari River, a subsidiary of the Rupen River. Being located closer to a river than Vaharvo Timbo, Loteshwar might have been favoured as a more permanent habitation site, which might explain why this site was occupied by hunter-gatherer groups for a longer period (ca. 2400 years, as opposed to the ca. 600 years in which Vaharvo Timbo was occupied according to AMS radiocarbon dates) and why Chalcolithic (Anarta) agropastorlists also inhabited this dune between ca. 3700 and 2200 years ago [53, 65–67]. However, the absence of studies related to the utilisation of specific tools currently restricts further inferences.

To summarise, the presence of microliths by itself should not be used to define an occupation as Mesolithic; considering the long time span and varied nature of such deposits. Intricate patterns of existence by various groups of people come to light only when an in-depth analysis of archaeological assemblages is carried out. More scientific excavations and secure dates are needed to better understand this period of Indian prehistory and the ways in which landscapes and environments were perceived and understood by the people who inhabited them. Such variability which needs to be explored thoroughly by all the future researchers to get a better understanding of microliths and their place in the hunter-gatherer/Mesolithic societies in western India. This study has established the nature and variability of the microlithic tool-kit of Holocene hunter-gatherer populations of western India, which can now be used as a reference for future hunter-gather research in this region as well as the neighbouring region of Sindh, Pakistan.

## Supporting information

S1 Appendix Outcome data of the statistical analyses. (DOCX)

S1 Table Basic raw data from Loteshwar and Vaharvo Timbo. (XLSX)

S2 Table Raw metric data from tools and lithic debitage Loteshwar. (XLSX)

S3 Table Raw metric data from tools and lithic debitage Vaharvo Timbo. (XLSX)
