# Supplementary material for: Microlithic variation and the Mesolithic occupations of western India
Source: PLoS One. 2022 Jun 22;17(6):e0267654. doi: 10.1371/journal.pone.0267654 (PMC9216571; doi:10.1371/journal.pone.0267654)
Supplement: S1 Appendix — (DOCX) [file pone.0267654.s001.docx]

> # Obliquely blunted blades

> wilcox.test(Breadth ~ Type, data=Ob_blades)

Wilcoxon rank sum test with continuity correction

data: Breadth by Type

W = 16, p-value = 0.044

alternative hypothesis: true location shift is not equal to 0

Warning message:

In wilcox.test.default(x = c(4.21, 5.26, 4.69, 5.33, 4.21, 4.66, :

cannot compute exact p-value with ties

> wilcox.test(Thickness ~ Type, data=Ob_blades)

Wilcoxon rank sum test with continuity correction

data: Thickness by Type

W = 32.5, p-value = 0.3747

alternative hypothesis: true location shift is not equal to 0

Warning message:

In wilcox.test.default(x = c(1.22, 1.14, 1.14, 1.16, 1.23, 1.71, :

cannot compute exact p-value with ties

> # Retouched blades

> wilcox.test(Breadth ~ Type, data=Retouched_blades)

Wilcoxon rank sum test with continuity correction

data: Breadth by Type

W = 155, p-value = 0.2333

alternative hypothesis: true location shift is not equal to 0

Warning message:

In wilcox.test.default(x = c(4.22, 5.71, 5.57, 6.36, 7.24, 3.77, :

cannot compute exact p-value with ties

> wilcox.test(Thickness ~ Type, data=Retouched_blades)

Wilcoxon rank sum test with continuity correction

data: Thickness by Type

W = 166, p-value = 0.3714

alternative hypothesis: true location shift is not equal to 0

Warning message:

In wilcox.test.default(x = c(1.52, 1.41, 1.9, 1.73, 2.23, 1.86, :

cannot compute exact p-value with ties

> # Backed blades

> wilcox.test(Breadth ~ Type, data=Backed_blades)

Wilcoxon rank sum test with continuity correction

data: Breadth by Type

W = 183.5, p-value = 0.001419

alternative hypothesis: true location shift is not equal to 0

Warning message:

In wilcox.test.default(x = c(4.25, 4.7, 3.94, 4.35, 5.07, 4.19, :

cannot compute exact p-value with ties

> wilcox.test(Thickness ~ Type, data=Backed_blades)

Wilcoxon rank sum test with continuity correction

data: Thickness by Type

W = 309.5, p-value = 0.2926

alternative hypothesis: true location shift is not equal to 0

Warning message:

In wilcox.test.default(x = c(1.09, 1.54, 1.82, 1.63, 2.18, 1.58, :

cannot compute exact p-value with ties

> # Simple blades

> wilcox.test(Breadth ~ Type, data=Simple_blades)

Wilcoxon rank sum test with continuity correction

data: Breadth by Type

W = 7005.5, p-value = 0.009719

alternative hypothesis: true location shift is not equal to 0

> wilcox.test(Thickness ~ Type, data=Simple_blades)

Wilcoxon rank sum test with continuity correction

data: Thickness by Type

W = 9370, p-value = 0.2115

alternative hypothesis: true location shift is not equal to 0

> # Points

> wilcox.test(Length ~ Type, data=Points)

Wilcoxon rank sum test

data: Length by Type

W = 127, p-value = 0.1592

alternative hypothesis: true location shift is not equal to 0

> wilcox.test(Breadth ~ Type, data=Points)

Wilcoxon rank sum test with continuity correction

data: Breadth by Type

W = 111.5, p-value = 0.4861

alternative hypothesis: true location shift is not equal to 0

Warning message:

In wilcox.test.default(x = c(4.96, 1.68, 4.61, 5.89, 4.91, 4.54, :

cannot compute exact p-value with ties

> wilcox.test(Thickness ~ Type, data=Points)

Wilcoxon rank sum test with continuity correction

data: Thickness by Type

W = 86.5, p-value = 0.676

alternative hypothesis: true location shift is not equal to 0

Warning message:

In wilcox.test.default(x = c(0.99, 2.22, 1.23, 3.33, 1.07, 1.13, :

cannot compute exact p-value with ties

> # Trapeziums

> wilcox.test(Length ~ Type, data=Trapeziums)

Wilcoxon rank sum test

data: Length by Type

W = 59, p-value = 0.2375

alternative hypothesis: true location shift is not equal to 0

> wilcox.test(Breadth ~ Type, data=Trapeziums)

Wilcoxon rank sum test

data: Breadth by Type

W = 40, p-value = 0.778

alternative hypothesis: true location shift is not equal to 0

> wilcox.test(Thickness ~ Type, data=Trapeziums)

Wilcoxon rank sum test

data: Thickness by Type

W = 25, p-value = 0.1288

alternative hypothesis: true location shift is not equal to 0

> # Lunates

> wilcox.test(Length ~ Type, data=Lunates)

Wilcoxon rank sum test with continuity correction

data: Length by Type

W = 494.5, p-value = 0.03232

alternative hypothesis: true location shift is not equal to 0

Warning message:

In wilcox.test.default(x = c(5.52, 10.56, 8.13, 7.32, 17.34, 14.21, :

cannot compute exact p-value with ties

> wilcox.test(Breadth ~ Type, data=Lunates)

Wilcoxon rank sum test with continuity correction

data: Breadth by Type

W = 362.5, p-value = 0.0004067

alternative hypothesis: true location shift is not equal to 0

Warning message:

In wilcox.test.default(x = c(4.83, 5.18, 4.18, 3.83, 5.59, 7.32, :

cannot compute exact p-value with ties

> wilcox.test(Thickness ~ Type, data=Lunates)

Wilcoxon rank sum test with continuity correction

data: Thickness by Type

W = 508.5, p-value = 0.04632

alternative hypothesis: true location shift is not equal to 0

Warning message:

In wilcox.test.default(x = c(2.12, 2.55, 1.48, 1.3, 2.98, 2.85, :

cannot compute exact p-value with ties

> # Scalene triangles

> wilcox.test(Length ~ Type, data=Scalene_triangles)

Wilcoxon rank sum test with continuity correction

data: Length by Type

W = 82, p-value = 0.03355

alternative hypothesis: true location shift is not equal to 0

Warning message:

In wilcox.test.default(x = c(8.87, 9.81, 9.23, 8.56, 10.75, 8.86, :

cannot compute exact p-value with ties

> wilcox.test(Breadth ~ Type, data=Scalene_triangles)

Wilcoxon rank sum test with continuity correction

data: Breadth by Type

W = 54, p-value = 0.6708

alternative hypothesis: true location shift is not equal to 0

Warning message:

In wilcox.test.default(x = c(5.18, 6.69, 4.57, 4.99, 6.76, 5.37, :

cannot compute exact p-value with ties

> wilcox.test(Thickness ~ Type, data=Scalene_triangles)

Wilcoxon rank sum test with continuity correction

data: Thickness by Type

W = 57, p-value = 0.5436

alternative hypothesis: true location shift is not equal to 0

Warning message:

In wilcox.test.default(x = c(1.05, 2.54, 1.02, 1.42, 1.83, 1.57, :

cannot compute exact p-value with ties

> # Isosceles triangles

> wilcox.test(Length ~ Type, data=Isosceles_triangles)

Wilcoxon rank sum test

data: Length by Type

W = 34, p-value = 0.2557

alternative hypothesis: true location shift is not equal to 0

> wilcox.test(Breadth ~ Type, data=Isosceles_triangles)

Wilcoxon rank sum test with continuity correction

data: Breadth by Type

W = 42, p-value = 0.5517

alternative hypothesis: true location shift is not equal to 0

Warning message:

In wilcox.test.default(x = c(4.1, 5.56, 5.31, 4.14, 3.65, 4.87, :

cannot compute exact p-value with ties

> wilcox.test(Thickness ~ Type, data=Isosceles_triangles)

Wilcoxon rank sum test with continuity correction

data: Thickness by Type

W = 47.5, p-value = 0.8336

alternative hypothesis: true location shift is not equal to 0

Warning message:

In wilcox.test.default(x = c(1.87, 1.96, 1.78, 1.62, 1.02, 1.76, :

cannot compute exact p-value with ties

> # Conical cores

> wilcox.test(Length ~ Type, data=Conical)

Wilcoxon rank sum test with continuity correction

data: Length by Type

W = 1026, p-value = 0.3366

alternative hypothesis: true location shift is not equal to 0

> wilcox.test(Breadth ~ Type, data=Conical)

Wilcoxon rank sum test with continuity correction

data: Breadth by Type

W = 858.5, p-value = 0.73

alternative hypothesis: true location shift is not equal to 0

> wilcox.test(Thickness ~ Type, data=Conical)

Wilcoxon rank sum test with continuity correction

data: Thickness by Type

W = 835.5, p-value = 0.5992

alternative hypothesis: true location shift is not equal to 0

> # Cylindrical cores

> wilcox.test(Length ~ Type, data=Cylindrical)

Wilcoxon rank sum test with continuity correction

data: Length by Type

W = 244, p-value = 0.8805

alternative hypothesis: true location shift is not equal to 0

Warning message:

In wilcox.test.default(x = c(12.68, 16.85, 16.16, 9.88, 24.19, 13.46, :

cannot compute exact p-value with ties

> wilcox.test(Breadth ~ Type, data=Cylindrical)

Wilcoxon rank sum test with continuity correction

data: Breadth by Type

W = 160, p-value = 0.1026

alternative hypothesis: true location shift is not equal to 0

Warning message:

In wilcox.test.default(x = c(12.91, 10.54, 6.51, 9.64, 12.24, 8.98, :

cannot compute exact p-value with ties

> wilcox.test(Thickness ~ Type, data=Cylindrical)

Wilcoxon rank sum test with continuity correction

data: Thickness by Type

W = 246, p-value = 0.8467

alternative hypothesis: true location shift is not equal to 0

Warning message:

In wilcox.test.default(x = c(7.68, 7.09, 8.85, 9.41, 9.45, 8, 9.79, :

cannot compute exact p-value with ties

> # Wedge cores

> wilcox.test(Length ~ Type, data=Wedges)

Wilcoxon rank sum test

data: Length by Type

W = 82, p-value = 1

alternative hypothesis: true location shift is not equal to 0

> wilcox.test(Breadth ~ Type, data=Wedges)

Wilcoxon rank sum test

data: Breadth by Type

W = 124, p-value = 0.03156

alternative hypothesis: true location shift is not equal to 0

> wilcox.test(Thickness ~ Type, data=Wedges)

Wilcoxon rank sum test with continuity correction

data: Thickness by Type

W = 147, p-value = 0.0008932

alternative hypothesis: true location shift is not equal to 0

Warning message:

In wilcox.test.default(x = c(11.55, 12.68, 11.63, 14.9, 9.75, 11.21, :

cannot compute exact p-value with ties

> # Block cores

> wilcox.test(Length ~ Type, data=Blocks)

Wilcoxon rank sum test

data: Length by Type

W = 15, p-value = 0.9273

alternative hypothesis: true location shift is not equal to 0

> wilcox.test(Breadth ~ Type, data=Blocks)

Wilcoxon rank sum test

data: Breadth by Type

W = 17, p-value = 0.6485

alternative hypothesis: true location shift is not equal to 0

> wilcox.test(Thickness ~ Type, data=Blocks)

Wilcoxon rank sum test

data: Thickness by Type

W = 20, p-value = 0.3152

alternative hypothesis: true location shift is not equal to 0
